# Effect, Mechanism, and Applications of Coding/Non-coding RNA m6A Modification in Tumor Microenvironment

**DOI:** 10.3389/fcell.2021.711815

**Published:** 2021-09-30

**Authors:** Chaohua Si, Chen Chen, Yaxin Guo, Qiaozhen Kang, Zhenqiang Sun

**Affiliations:** ^1^Department of Colorectal Surgery, The First Affiliated Hospital of Zhengzhou University, Zhengzhou, China; ^2^School of Life Sciences, Zhengzhou University, Zhengzhou, China; ^3^Henan Academy of Medical and Pharmaceutical Sciences, Zhengzhou University, Zhengzhou, China; ^4^School of Basic Medical Sciences, Zhengzhou University, Zhengzhou, China

**Keywords:** tumor microenvironment, m6A RNA modification, coding RNA, non-coding RNA, cancer

## Abstract

The tumor microenvironment (TME), which includes immune cells, fibroblasts, and other components, is the site of tumor cell growth and metastasis and significantly impacts tumor development. Among them, N6-methyladenosine RNA modifications (m6A RNA modifications) are the most abundant internal modifications in coding and non-coding RNAs, which can significantly influence the cancer process and have potential as biomarkers and potential therapeutic targets for tumor therapy. This manuscript reviews the role of m6A RNA modifications in TME and their application in tumor therapy. To some extent, an in-depth understanding of the relationship between TME and m6A RNA modifications will provide new approaches and ideas for future cancer therapy.

## Introduction

In recent years, cancer is still one of the most important diseases affecting human life and health. As cancer research has progressed, we found that the tumor microenvironment (TME) also profoundly influences the course of tumor development in addition to the tumor cell (Hinshaw and Shevde, 2019). TME is a dynamic, complex system of multiple components, including immune cells, fibroblasts, and lymphocytes, that play a crucial role in cancer development and progression, and its interaction with tumor cells is a critical factor in the success of tumor cell-tolerant immune escape (Ocana et al., 2019). Meanwhile, we have known that hundreds of chemical modifications occur in TME, which play different roles in TME at different stages of cancer development. In addition, N6-methyladenosine RNA modifications (m6A RNA modifications) are the most abundant modification in TME, and the existing studies have shown that m6A RNA modifications occurring in TME are a vital factor mediating tumor progression and influencing tumor treatment outcome (Chen X. Y. et al., 2019; Cheng H. S. et al., 2019; Wang J. et al., 2020). Therefore, enhanced knowledge and understanding of TME may lead to new ideas and approaches for treating cancer patients.

Starting with Crick’s central principle 60 years ago, plenty of previous studies have focused on coding RNA, which make up about 2% of the human genome sequence but are vital to humans (Crick, 1970; Poller et al., 2018). However, non-coding RNA, which accounts for 98% of the human genome, has been ignored as “junk sequences” (Fabbri et al., 2019). Furthermore, with technology development, such as high-throughput sequencing technology, non-coding RNA has entered people’s field of vision with a new role. As the research goes further, we classified non-coding into microRNA, lncRNA, circRNA, and so on (Matsui and Corey, 2017; Momen-Heravi and Bala, 2018). Non-coding RNA lacks the potential to encode proteins or peptides but has a high degree of transcriptional activity, which plays an essential role in gene regulation, structure and performs biological functions at the RNA level (Wong et al., 2018). Recent studies have shown that aberrant expression of non-coding RNA is ubiquitous in different types of cancers, revealing that it may play an essential role in human cancers.

As the most abundant modification in TME, m6A RNA modification is one of the critical factors affecting the biological function of RNA (Ma et al., 2019; Yang G. et al., 2020). In general, m6A RNA modification requires the involvement of three factors: m6A methylase (writer), m6A demethylase (eraser), and m6A binding protein (reader) (Shi et al., 2019). It usually influences cancer progression by regulating biological functions associated with cancer, including proliferation, metastasis, stem cell differentiation, and stabilization (Roskoski, 2019). Currently, m6A RNA modifications are also increasingly used to detect and diagnose cancer (Niu et al., 2019).

This review mainly outlines the links between coding/non-coding RNA and m6A RNA modification in the TME. Meanwhile, we describe the implications of the interactions between the three for the biological functions that influence the cancer process and discuss their possible future role in clinical applications.

## Tumor Microenvironment

Tumor microenvironment consists mainly of an immune microenvironment dominated by immune cells and a non-immune microenvironment dominated by fibroblasts, formed by the combined action of malignant tumor cells and non-transformed cells (Balkwill et al., 2012; Chen and Hambardzumyan, 2018; Costa et al., 2018; Baghban et al., 2020). To date, most of our research has focused on the immune microenvironment, and the results have shown that alterations in TME profoundly influence tumorigenesis and progression, not only by causing tumor heterogeneity but also by influencing patient resistance (Pottier et al., 2015; Wang J. J. et al., 2018). For example, in breast cancer, one of the mechanisms of action of TME is the removal or alteration of tumor components, thereby impeding anti-tumor immunity (Jung et al., 2016). In liver cancer, we predict tumor progression based on the dynamics of TME. At the same time, the stromal cells in TME can influence the invasion and migration of tumor cells (Yang et al., 2011). Thus, TME can be used as a therapeutic target for cancer and as a signal to detect cancer progression (Jarosz-Biej et al., 2019).

**FIGURE 1:**
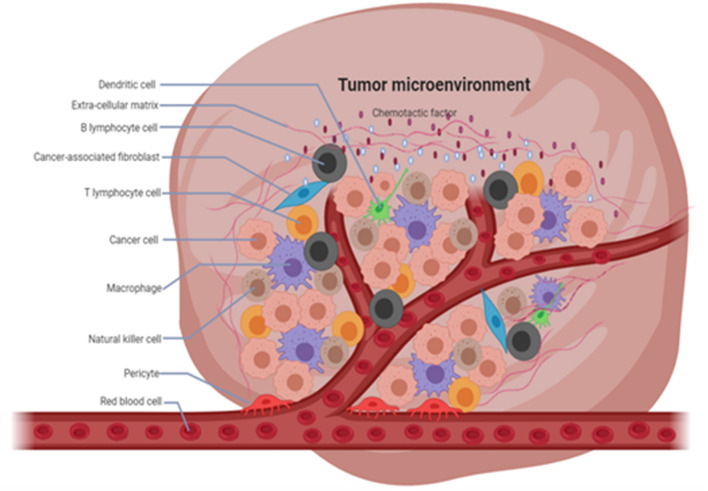
Composition of tumor microenvironment (TME). TME has a complex composition, consisting of immune cells, fibroblasts, and other components which play an important role in the development of cancer.

At the same time, TME, as a metabolic site for the growth of numerous cells, is rich in metabolites such as succinic acid, D-2HG, and fumaric acid, which are essential for the normal development of the cells and the organism. These metabolites can act as cofactors or antagonists of epigenetic modification enzymes, affecting numerous epigenetic processes such as m6A RNA modification (Zhao Y. et al., 2020). METTL3 and METTL14 are important components of the m6A modifying enzyme complex, and they can form a stable dimer involved in the methylation process, which is dynamically regulated by substrates and metabolites. S-adenosylmethionine (SAM/AdoMet) acts as a universal intracellular methyl donor. However, because METTL14 does not have a SAM binding site, only METTL3 is active, but METTL14 makes an important contribution in binding substrates, where SAM is produced by a carbon metabolic pathway consisting of the folate and methionine cycles (Sledz and Jinek, 2016; Wang et al., 2016). Similarly, metabolites within TME can influence the demethylation of m6A RNA represented by FTO and ALKBH5, such as 2-oxoglutarate, a key metabolite of the citric acid cycle. If this product is mutated, the demethylation of FTO and ALKBH5 is drastically reduced or even completely lost. In addition, 2-oxoglutarate can also undergo a series of biological reactions to convert to fumaric acid, and due to their structural similarity, these metabolites can often bind to competing products of 2-oxoglutarate become m6A demethylase inhibitors (Feng et al., 2014; Zhang et al., 2019; Figure 1).

## M6A RNA Modification

**FIGURE 2:**
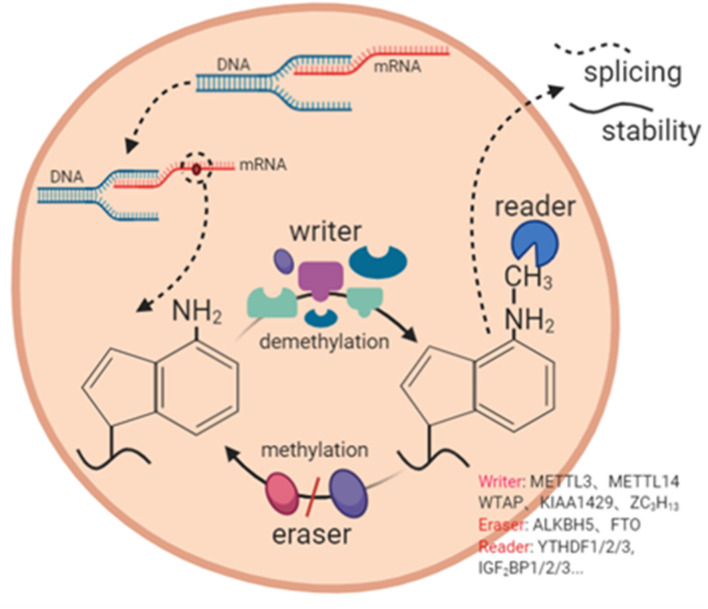
The mechanism of m6A RNA modification. m6A RNA modification is a dynamic and complex process with the participation of m6A methylase (writer), m6A demethylase (eraser), and m6A binding protein (reader). It participates in RNA splicing, RNA stability and other processes.

To date, hundreds of chemical modifications have been reported, among which m6A RNA modification is the most prevalent in coding and non-coding RNA (Ding et al., 2018; Wang et al., 2019a). m6A RNA modification is a dynamic and reversible process (Wang Q. et al., 2020), it requires the involvement of an m6A modifies enzyme complexes (Lin et al., 2016). In recent years, m6A RNA modification has made great progress in regulating RNA transcription (Wang J. et al., 2020), splicing (Ma et al., 2019), translation (Liu et al., 2018c), and stability (Panneerdoss et al., 2018). Below we summarize the relationship between m6A RNA modification and the above biological processes (Figure 2).

## M6A RNA Modification and RNA Splicing

The DNA template strand contains introns, and RNA splicing removes introns and joins exons to form a continuous RNA molecule. mRNA splicing produces mRNAs that encode information required for growth and development (Zhao et al., 2014). FTO, a component of the m6A modifying enzyme complex, is responsible for the regulation of exon splicing in the 3′ and 5′ exon regions. Zhao et al. showed that the expression level of FTO was negatively correlated with the level of m6A RNA modification during adipogenesis and that the two could interact to influence the development and treatment process of cancer patients.

## M6A RNA Modification and RNA Transcription

RNA transcription is the process of RNA polymerase catalyzing RNA synthesis from a template strand of DNA and four nucleotides, i.e., the transfer of biological information from DNA to RNA, guided by the principle of complementary base pairing (Zhao W. et al., 2020). The m6A RNA modification takes part in and affects the transcriptional process of RNA. KIAA1429 is one of the important components of the m6A modifying enzyme complex and participated in the composition of the m6A writer. Lan et al. showed that in the absence of interference by KIAA1429, the interaction between HuR and GATA3 pre-mRNA put them in dynamic equilibrium in hepatocellular carcinoma again. In contrast, the addition of KIAA1429 affected the progression of tumor development by preferentially inducing m6A methylation on the 3′ UTR of GATA3 pre-mRNA in hepatocellular carcinoma cells, followed by the isolation of HuR and degradation of GATA3 pre-mRNA, and finally, the down-regulation of GATA3 expression (Lan T. et al., 2019).

## M6A RNA Modification and RNA Translation

Proteins are the leading performers of biological functions, and RNA translation belongs to the second part of protein biosynthesis, the first part being RNA transcription. According to the central law, RNA translation is deciphering the base sequences of mature messenger RNA molecules to produce specific amino acids. METTL3, which selectively enhances or inhibits mRNA translation, is one of the significant components of m6A methyltransferase (Choe et al., 2018). Meanwhile, insulin-like growth factor 2 mRNA-binding proteins (IGF2BPs) are important components of the m6A-modifying enzyme complex and act as “readers.” Huang et al. (2018) showed that IGF2BP protein recognizes m6A RNA modifications and enhances mRNA stability and translation.

## M6A RNA Modification and RNA Stability

The m6A RNA modification has the function of regulating the stability of RNA, which refers to the ability of RNA to maintain its original structure or resist degradation when external conditions or other factors change (Yang L. et al., 2020). The YTH domain family, which includes YTH domain family proteins 1-3 (YTHDF1-3) and YTH domain-containing proteins 1-2 (YTHDC1-2), i.e., the DF family and DC family, is one of the ‘reader” components of the m6A modifying enzyme complex and plays an important function in RNA stability. For example, YTHDF2, as one of the readers of m6A modification, can promote mRNA degradation by recruiting the CCR4-NOT deadenylase complex, thereby reducing the stability of the targeted transcript (Wang et al., 2014; Du et al., 2016). Moreover, Huang et al. (2018) showed that binding of m6A-modified RNA to corresponding binding proteins also could affect RNA stability.

## M6A RNA Modifications Can Regulate Biological Functions

**FIGURE 3:**
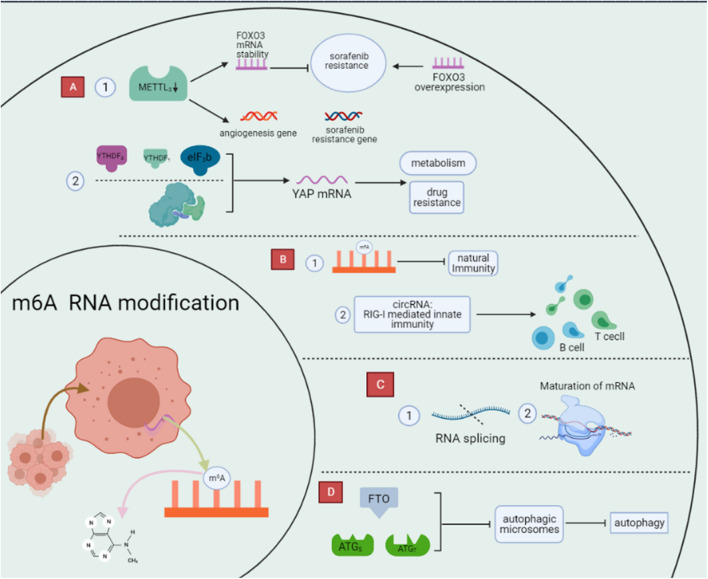
Regulation of biological function of m6A RNA modification. m6A modifications are the most abundant modifications within RNA and can profoundly influence the process of cancer development by regulating biological functions such as drug resistance, immune metabolism, and autophagy through a variety of pathways. **(A)** m6A RNA modification affect tumor drug resistance. **(B)** m6A RNA modification affect immunity. **(C)** m6A RNA modification affect metabolism. **(D)** m6A RNA modification affect autophagy.

m6A RNA modification’s ability to affect cancer is proven in various cancers, and it is likely to be a marker for the molecular diagnosis of tumors. Meanwhile, deepening the understanding of the effects of m6A RNA modification on cancer may provide new targets and ideas for researching and developing clinical molecular targeted therapies (Dai et al., 2018; He et al., 2019; Lan Q. et al., 2019; Zhou et al., 2020). In the following, taking gastric cancer as an example, METTL3 can promote the development and progression of gastric cancer by mediating m6A RNA modification of HDGF mRNA (Wang Q. et al., 2020). In contrast, in hepatocellular carcinoma, Chen and Wong (2020) showed that m6A RNA modification regulates the expression of different downstream targets by regulating mRNA stability and translation efficiency (Figure 3).

## M6A RNA Modification Affect Tumor Drug Resistance

We usually use several approaches in cancer treatment, among which targeted therapies have received more and more attention because of their more significant effectiveness (Li B. et al., 2020). Although targeted therapies are advancing rapidly, they still face tumor resistance that severely affects the efficacy of cancer treatment (Lin et al., 2020). m6A RNA modifications are the most common RNA modifications that can affect RNA splicing, degradation, and translation by regulating cell proliferation, metabolism, metastasis, and ultimately tumor drug resistance (Huang et al., 2018). For example, Liu et al. showed that RNA m6A modification specifically regulates sorafenib resistance in hepatocellular carcinoma through FOXO3-mediated autophagy. The mechanism is that METTL3 depletion under hypoxic conditions promotes sorafenib resistance and angiogenic genes in HCC cells cultured *in vitro* and subsequently activates the autophagic pathway (Liu et al., 2020b). METTL3 is significantly downregulated in human sorafenib-resistant hepatocellular carcinoma, whereas METTL3 depletion markedly enhances FOXO3 mRNA stability and eliminates METTL3 mediated sensitivity to sorafenib m6A dependence, which could restore resistance to HCC if FOXO3 overexpression was present.

Furthermore, Jin et al. showed that m6A RNA methylation promotes YAP translation and increases YAP activity, thereby inducing drug resistance and metastasis in NSCLC. The mechanism is that m6A methylation causes YTHDF1/3 and eIF3b to join the translation initiation complex, thereby promoting the translation of YAP mRNA. Also, m6A RNA methylation improves YAP mRNA stability through the MALAT1-miR-1914-3p-YAP axis (Jin et al., 2019).

## M6A RNA Modification Affect Immunity

The immune system is vital to the body and helps us to defend ourselves against many harmful microorganisms. Recent studies have shown that m6A RNA modification affects the immune system and the function of immune cells to a certain extent, the more obvious of which is that deletion of METTL3 will lead to impaired maturation of these cells in response to lipopolysaccharides and decreased expression of CD40 and CD80, thus inducing a decrease in T cell responsiveness. Also, m6A RNA modification can affect the development of the immune system. In a mouse model, high expression of METTL3 is required for the differentiation of blood-derived endothelial cells into hematopoietic stem cells (Shulman and Stern-Ginossar, 2020). What is more, immune dysregulation is an important cause of several immune diseases, including systemic lupus erythematosus (SLE) and rheumatoid arthritis. It has been reported that m6A RNA modifications can regulate RNA and other gene expressions in immune cells, affecting SLE immune cell pathogenesis (Ma et al., 2019; Sekar and Lakshmanan, 2020). Meanwhile, m6A RNA modifications in circRNA play an essential role in tumor development and anti-tumor immunity (Chen Y. G. et al., 2019). Exogenous circRNA can effectively stimulate immune signaling, and m6A RNA modifications are the main contributors to the immune effects of circRNA. For example, exogenous circRNA activates RIG-I-mediated innate immunity, induces activation of antigen-specific B and T cells, and anti-tumor activity *in vivo* (Poller et al., 2018). In addition, m6A-modified RNAs are degraded by YTHDF2, suppressing natural immunity. These results indicate that m6A RNA modification plays an essential regulatory role in the tumor immune process (Paramasivam and Vijayashree Priyadharsini, 2020). Therefore, a deeper understanding of the relationship between m6A RNA modifications and immunity may open new doors to treat immune diseases.

## M6A RNA Modification Affect Metabolism

Epigenetic regulation of organisms is a complex process. m6A RNA modification is the most prevalent modification in eukaryotic cells and represents a new trajectory of epigenetic modifications. It plays a vital role in regulating RNA metabolisms, such as regulating splicing and translation. One way of RNA editing is converting A to I with the involvement of RNA adenosine deaminase (ADAR). Reports suggest that A-to-I is a crucial factor affecting RNA metabolism and that m6A RNA modification is inversely correlated with A-to-I. A possible reason for this is that m6A RNA modification affects RNA structure (Torsin et al., 2021).

Meanwhile, *in vivo* immunofluorescence analysis revealed that METTL3, a component of the m6A methyltransferase complex, is located on the mRNA splicing factor, revealing that m6A RNA modifications may play a regulatory role in RNA metabolism and have an impact on cellular reprogramming (Yang et al., 2018). As the study progressed, Liu Y. et al. (2019) found that m6A methylation affected multiple aspects of mRNA metabolism, from expression in the nucleus to processing of pre-mRNA to attenuation of the translational machinery of mRNA in the cytoplasm. The transition from pre-mRNA to mature mRNA requires a splicing process, and there is evidence that m6A RNA modification is a vital splicing regulator (Liu et al., 2014; Ping et al., 2014). First, based on PAR-CLIP analysis, it was observed that mRNAs with selective splicing have more METTL3 binding and methylation sites. A recent study also showed that METTL3 could play a role in spermatogenesis by initiating selective splicing of related mRNAs to regulate sperm differentiation and meiosis (Dominissini et al., 2012). Secondly, m6A erasers and readers also differentially affect RNA splicing, with FTO binding to pre-mRNAs in the nucleus to initiate selective spliced exons and YTHDF1 binding directly to m6A-modified variable splice exons to facilitate their incorporation into mRNAs (Meyer et al., 2012; Bartosovic et al., 2017).

## M6A RNA Modification Affect Autophagy

Autophagy is a complex biological process that involves the engulfment of organelles and proteins by autophagosomes (Doria et al., 2013; Galluzzi et al., 2017; Russo and Russo, 2018), followed by digestion by lysosomes, and finally, entry and recycling of cellular processes (Kimura et al., 2007; Yuan et al., 2020). It can influence multiple aspects of cancer, such as regulating resistance to sorafenib in hepatocellular carcinoma. The mechanism is the downregulation of the m6A demethylase component FTO, which affects the expression of ATG5 and ATG7, ultimately reducing the ability of autophagic microsomes and inhibiting autophagy. That is, after FTO Silencing, YTHDF2, modified with high levels of m6A RNA, binds to ATG5 and ATG7 transcripts, and mRNA degradation increases, leading to reduced protein expression and affecting the autophagic process (Jin et al., 2018; Wang C. Y. et al., 2020, Wang X. et al., 2020).

## M6A RNA Modification Affects the Infiltration Characteristics of Cancer Cells in Tumor Microenvironment

### m6A RNA Modification Can Activate Oncogenes Within Tumor Microenvironment

m6A RNA modifications can influence cancer development by activating tumor-associated genes (He L. et al., 2018; Zheng W. et al., 2019). Depending on the outcome, we can classify m6A RNA modification into promoter and suppressor effects. Next, we will specifically summarize the dual role of m6A RNA modification in cancer.

On the one hand, m6A RNA modification can promote tumorigenesis and development. Take bladder cancer, lung cancer, acute myeloid leukemia, ovarian cancer, breast cancer, pancreatic cancer, colorectal cancer, and nasopharyngeal cancer. Gu et al. showed that in bladder cancer, the expression of METTL3, METTL14, and the oncogene CDCP1 influenced the progression of tumor development. In bladder cancer, m6A expression showed upregulation, and inhibition of METTL3 inhibited proliferation, invasion, and migration of bladder cancer tumor cells. In addition, METTL14 expression is downregulated in bladder cancer, and some studies have shown that eliminating METTL14 promotes capsule proliferation (Gu et al., 2019). In lung cancer, METTL3 expression increases. In a mouse model, deletion of METTL3 promoted apoptosis in transplanted tumor cells. Related experiments showed that METTL3 expression levels in A549 cells responded to the induction of simvastatin. Chen et al. (2020) showed that METTL3 positively regulates the level of EZH2 and modifies its mRNA via m6A, thereby affecting tumor progression (Choe et al., 2018). In acute myeloid leukemia, increased expression of METTL3 promotes the translation of SP1, which regulates the expression of the oncogene C-MYC, and METTL14 enhances the translation of MYB and MYC by maintaining its high expression (Barbieri et al., 2017; You et al., 2017). In ovarian cancer, increased expression levels of IGF2BP1 enhanced the expression of SRF and inhibited its degradation, thereby increasing the expression levels of SRF, FOXK1, and PDLIM7 (Ro, 2016; Muller et al., 2018, 2019). Meanwhile, METTL3 promotes growth, invasion, and migration in ovarian cancer by stimulating peripheral mRNA transfer and epithelial-mesenchymal transition. In breast cancer, increased expression levels of METTL3 would promote HBXIP expression and thus affect breast cancer phenotype. In addition, upregulation of ALKBH5 expression promotes mRNA stability and expression of the pluripotent silver NANOG gene, affecting breast cancer development (Deng et al., 2018). In pancreatic cancer, METTL3 regulates tumor progression and drug resistance through MAPK cascade, RNA splicing, and cellular regulation, and therefore further studies METTL3’s interaction with these processes is essential to understand the functional mechanisms of METTL3 (Taketo et al., 2018). The results of Song et al. (2020) showed increased expression of the YTHDF1 gene in colorectal cancer, where activation of the WNT/β-catenin signaling pathway may initiate transcription-dependent oncogenic effects, promote gross cell cycle progression, and regulate resistance. Knockdown of the YTHDF1 gene would affect the activity of the WNT/β-catenin signaling pathway and inhibit the tumorigenicity of colorectal cancer cells (Nishizawa et al., 2018; Bai et al., 2019). Multiple research teams have found that Nasopharyngeal carcinoma m6A RNA modification affects the overall tumorigenicity of FAM225A and ultimately affects the proliferation of nasopharyngeal carcinoma tumor cells. In addition, METTL3 promotes the expression of EZH2 protein by mediating m6A modification of EZH2 mRNA, which increases the malignancy of nasopharyngeal carcinoma cells by silencing CDKN1C, thereby affecting the development and progression of nasopharyngeal carcinoma (Lai et al., 2018; Wang et al., 2019b; Zheng Z. Q. et al., 2019; Meng et al., 2020).

**FIGURE 4:**
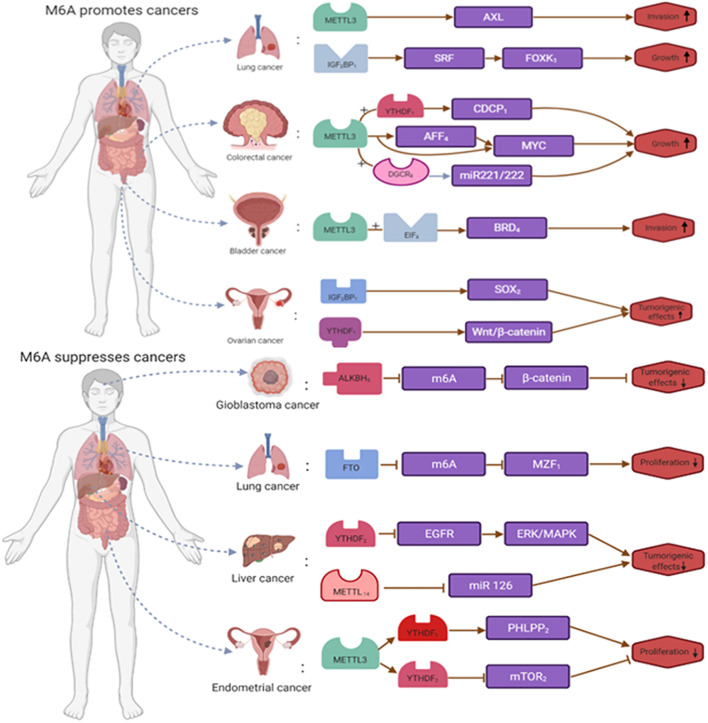
The dual roles of m6A RNA modification in cancer. m6A is modified to regulate the expression of tumor-related genes. m6A RNA modification promote the occurrence and development of tumors by enhancing the expression of tumor-related genes or inhibit the occurrence and development of tumors by inhibiting the expression of tumor-related genes.

On the other hand, m6A RNA modification inhibits tumorigenesis and progression. We take endometrial cancer, hepatocellular carcinoma, breast cancer, lung squamous cell carcinoma, and glioblastoma. In endometrial tumors, METTL3 expression decreased, and METTL14 mutated to some extent. m6A methylation reduction promoted cell proliferation, clone formation, migration, and invasion, affecting endometrial tumorigenesis and progression. In addition, FTO catalyzes the demethylation of HOXB13 mRNA in the 3′UTR region, thereby eliminating the recognition of YTHDF2 protein-modified m6A that affects tumor metastasis (Zhang L. et al., 2020). In hepatocellular carcinoma, Hou et al. (2019) showed a unique m6A-mRNA editing process in the hypoxic state. m6A RNA modification through YTHDF2-mediated EGFR degradation plays a role in tumor suppression. In addition, YTHDF2 inhibits ERK/MAPK signaling by destabilizing EGFR mRNA and affecting hepatocellular carcinoma progression (Ma et al., 2017). In breast cancer, Zhang et al. (2016a,b) showed that ALKBH5 expression increases, that NANOG can maintain and regulate tumor stem cells, and that increased ALKBH5 expression correlates with decreased NANOG mRNA m6A levels increased mRNA stability. In lung squamous cell carcinoma, the expression level of FTO is lower. By inhibiting m6A methylation, FTO enhances mRNA stability, promotes the expression of the oncogene MZF1, and affects the progression of lung squamous carcinoma. In addition to FTO, METTL3 expression was also abnormal in lung squamous cell carcinoma (Liu et al., 2018b; Cayir et al., 2019). Wang M. et al. (2020)) showed that ALKBH5 expression increases in glioblastoma and that reduced m6A methylation promoted the expression of the oncogene FOX1, enhancing the self-renewal ability and tumorigenicity of glioblastoma stem cells (Zhang et al., 2017; Figure 4).

### m6A RNA Modification Can Activate Immune Cells

m6A RNA modifications can influence cancer development by activating immune cells. For example, immune checkpoint blockade therapy is a revolutionary change in cancer treatment, but many patients do not respond to or are resistant to immune checkpoint blockade therapy (Zhang et al., 2017). ALKBH5 is an important component of the m6A modifying enzyme complex, and it has been reported that deletion of the m6A demethylase ALKBH5 can make patients more sensitive to cancer immunotherapy (Fang et al., 2020). The mechanism is that ALKBH5 affects the efficacy of immunotherapy during immune checkpoint blockade by regulating the expression and lactate content of MCT4/Slc16a3 in TME and the composition of tumor-infiltrating T cells and myeloid suppressor cells. Among these, growth factor-α is a target gene for ALKBH5, MCT4/Slc16a3 and is involved in regulating extracellular lactate concentration, regulatory T cells, and MDSC aggregation in TME (Li N. et al., 2020).

### m6A RNA Modification Can Suppress Immune Cells

In addition to activating immune cells, m6A RNA modifications can also affect the cancer process by suppressing the expression of immune cells. CD4 regulatory T cells are involved in and resolve immune suppression in TME. The transcription factor Foxp3, a marker molecule of Treg cells, is regulated by the Suppressor of cytokine signaling (SOCS) family and activates STAT5 (Tong et al., 2018). It has been reported that in a mouse model that has been constructed with METTL3 deletion, elevated expression of the SOCS gene compared to controls inhibited IL2-STAT5 signaling pathway and maintained the inhibitory function of Treg (Lou et al., 2021). Secondly, in hepatocellular carcinoma, METTL3 overexpression then inhibited the expression of suppressor of cytokine signaling factor 2 (SOCS2) through an m6A-YTHDF2-dependent mechanism, which ultimately promoted tumor growth (Tuncel and Kalkan, 2019).

## The Roles of M6A RNA Modification in Tme of Various Cancers

**FIGURE 5:**
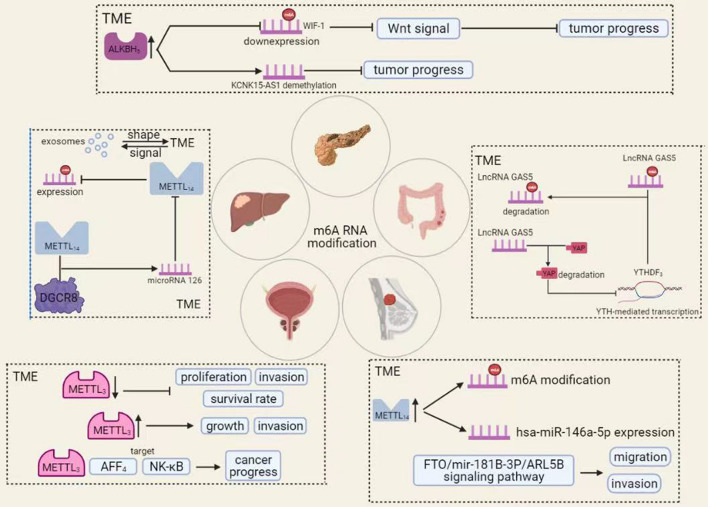
The roles of m6A RNA modification in TME of various cancers. A variety of chemical modifications occur within the tumor microenvironment that is important for tumorigenesis and progression. Among these, m6A RNA modification is the most abundant. In different tumors, m6A RNA modifications can affect cancer progression in various ways.

m6A RNA modifications found in TMEs of different cancers influence the onset and progression of cancer. In this review, we have selected several cancers and summarized the role of m6A RNA modifications in TME (Figure 5 and Table 1).

**TABLE 1:** Tlie role of m6A methylation in a variety of cancers.

Type	Molecular	Cancer	Role in cancer	Mechanism	Related targets	Function	References
		Leukemia	Oncogene	Promoting the translation	c-MYC, BCL2, and PTEN	Inhibit and induce differentiation, promote cell growth, and thus delay the occurrence of Leukemia	Visvanathan et al., 2018
			Oncogene	Enhancing the mRNA stability	SOX2	Reduced sensitivity to γ radiation	Chen et al., 2018
m6A writer	METTL3	Glioblastoma	Anti-oncogene	Regulating oncogenes	ADAM19, EPHA3, and KLF4	Inhibit the formation, growth, and development of glioblastoma	Cui et al., 2017
				Promote the translation	EGFR, TAZ, MAPK2(MK_2_), DNMT3A	Promotes growth, invasion, and survival	Choe et al., 2018
		Lung cancer	Oncogene	Enhancing the translation	BRD4	Promote tumor growth	Choe et al., 2018
		Liver cancer	Oncogene	Regulating target	SOCS2	Promotes cell proliferation and migration	Liu et al., 2018a
		Bladder cancer	Oncogene	Promoting the translation	CPCP1	Promote malignant transformation of cells	Vu et al., 2017
		Ovarian carcinoma	Oncogene	Promoting the translation	AXL	Promote cell proliferation Inhibition of cell	Liu et al., 2014
m6A writer	METTL3	Endometrial cancer	Anti-oncogene	Stimulating AKT activation	PHLPP2	Inhibition of cell proliferation, invasion and migration	Weng et al., 2018
		Breast cancer	Oncogene	Promoting the expression Enhancing	HBXIP	Promote cell proliferation	Wang et al., 2017
		Acute myeloid leukemia	Oncogene	m6A RNA modification and gene expression	SOCS2	Promote tumor growth	Vu et al., 2017
		Leukemia	Oncogene	Regulating mRNA stability and translation	SPI1	Inhibit the differentiation of leukemia cells and promote the self-renewal of stem	Wang et al., 2016
		Glioblastoma	Oncogene	Regulating oncogenes	ADAM19, EPHA3, KLF4	Promote tumor cell growth and self-renewal	Cui et al., 2017
m6A writer	METTL14	Endometrial cancer	Anti-oncogene	Stimulating AKT activation	PHLPP2	Inhibition of cell, proliferation, invasion, and migration	Weng et al., 2018
			Anti-oncogene	Regulating the miRNA processing	DGCR8	*In vivo* inhibits cell invasion and migration, and inhibits tumor growth	Bansal et al., 2014
		Hepatoma	Oncogene	Regulating its target	SOCS2	Promote cell proliferation	Tang et al., 2018
		Acute myeloid leukemia	Oncogene	Promoting tumorigenesis	MYC and MYB	Promote tumor growth	Weng et al., 2018
		Glioblastoma	Oncogene	Promoting expression	FOXM1	Promote cell proliferation	Bansal et al., 2014
		Breast cancer	Oncogene	Strengthening mRNA stability	NANOG	Increase the number of tumor stem cells	Zheng et al., 2013
m6A eraser	Alkbh5	Glioblastoma	Oncogene	Promoting expression	FOXM1	Promote cell proliferation	Zhang et al., 2017
		Acute myeloid leukemia	Anti-oncogene	Los of ALKBH5 copy number	ALKBH5	Decrease the copy number of ALKBH5	Kwok et al., 2017
		Pancreatic cancer	Anti-oncogene	Reducing methylation	KCNK15-AS1	Reduces methylation and inhibits tumor cell metastasis	He et al., 2018
		Glioblastoma	Anti-oncogene	Regulating oncogenes	ADAM19, EPHA3, and KLF4	Inhibit growth and tumor formation	Cui et al., 2017
		Leukemia	Oncogene	Regulating expression	ASB2 and RARA	It promotes the transformation of AML cells and inhibits cell differentiation	Li et al., 2017
m6A eraser	FTO	Lung cancer	Oncogene	Promoting the stability	MZF1	Promote the growth of tumor cells	Mauer et al., 2017
		Cervical squamous cell carcinoma	Oncogene	Regulating expression	β-catenin	Promote resistance to radiation therapy	Zhou et al., 2018
				Promoting tumorigenesis	ASB2 and RARA	Reduced m6A levels in transcripts	Li et al., 2017
		Acute myeloid leukemia	Oncogene	Promoting proliferation	MYC and CEBPA	Inhibit tumor cell proliferation and survival	Su et al., 2018
	IGF2BPs	Ovarian and Liver cancer	Oncogene	Enhancing mRNA stability	SRF	Promote tumor cell growth and cell invasion	Su et al., 2018
m6A reader	YTHDF1	Melanoma and colon cancer	Oncogene	Promoting the expression	lysosomal proteases	Promote tumor growth	Zheng et al., 2013
		Liver cancer	Oncogene	Regulating its target	SOCS2	Promotes cell proliferation and migration	Liu et al., 2018a
	YTHDF2	Pancreatic cancer	Anti-oncogene	Promoting growth and migration	m6A-containing mRNA	Promote tumor cell growth and cell invasion	Su et al., 2018

### Colorectal Cancer

Colorectal cancer is the third most deadly cancer in the world (Bray et al., 2018). Currently, we have made some progress in the diagnosis and treatment of this disease, but the prognosis of patients with advanced cancer remains poor (Nishihara et al., 2013). As research into cancer has intensified, TME has gradually attracted attention and attempted to use it in cancer treatment, in addition to malignant tumor cells. TME is the site of growth and transformation of malignant tumor cells, in which it produces and secretes various chemokines and growth factors that promote tumor development and progression (Bahrami et al., 2018). TME contains numerous immune components that can influence tumor development. It is part of the TME and is a very promising therapeutic target. The resulting tumor immune microenvironment has a dynamic character during tumor progression, in which many kinds of cells are involved (Zhang Y. et al., 2020). Ni et al. showed that disruption of the YAP signaling pathway significantly promotes the development and progression of colorectal cancer. The mechanism is that long-stranded non-coding RNA Gas5 is negatively regulated by the m6A reader YTHDF3, forming an adverse regulatory pathway through phosphorylation and degradation of YAP, thereby inhibiting the colorectal carcinogenesis process (Kino et al., 2010; Ni et al., 2019).

Meanwhile, a study by Xu et al. showed that the expression level of long-stranded non-coding RNA SATB2-AS1 was significantly increased in colorectal cancer. This study found that SATB2-AS1 interacted with chromatin regulatory proteins WDR5 and GADD45A in colorectal cancer cell species, with WDR5 catalyzing Lys4 trimethylation and affecting transcription when bound to methylated H3K4. SATB2-AS1 suppressed tumor metastasis in colorectal cancer by regulating STAB2, i.e., regulating cancer progression by affecting the tumor immune microenvironment (Xu et al., 2019).

### Hepatocellular Carcinoma

Hepatocellular carcinoma (HCC) is a common malignancy with high morbidity and mortality worldwide (Zhang et al., 2018). Patients with advanced hepatocellular carcinoma prognosis are poor under existing conditions, and new approaches are urgently needed. In recent years, immunotherapy has gained prominence in cancer treatment. Because TME is more variable at different stages of tumor development, it shows more significant variation in other individuals or distant locations of the same individual (Quail and Joyce, 2013). Immune cells can inhibit tumor development, but when they interact with specific cells of TME, the tendency for tumor development to spread will be more pronounced (Zamarron and Chen, 2011). Secondly, HCC can classify into multiple types that share a similar TME, both of which promote immune tolerance and immune escape through various mechanisms (Fu et al., 2019). TME is an essential partner of tumor cells, providing the necessary conditions for the development and progression of tumor cells, such as angiogenesis and tumor formation. Exosomes are information carriers for TME and are the molecular entities involved in building TME (Wu et al., 2019). In hepatocellular carcinoma, exosomes shape TME, provide energy, promote tumor growth, and induce angiogenesis. TME contains exosomes regulating cell line survival and development by giving stimulatory or inhibitory signals. The role of m6A RNA modifications, which occur mainly in TME in HCC, has attracted increasing attention (Bissell and Hines, 2011). For example, microRNA 126 is an essential component of the methyltransferase complex and decreased expression *in vitro* and *in vivo* (Ma et al., 2017). MicroRNA 126 is a significant component of METTL14 in HCC. METTL14 can influence the progression of tumor development by m6A-dependent regulation of PRI-microRNA 126 progression, leading to reduced expression of microRNA 126 (Hu et al., 2019).

### Bladder Cancer

Bladder cancer is one of the three significant urinary system tumors and is the most common malignancy of the urinary system, with the highest incidence among malignancies (Han et al., 2019). Bladder cancer develops as a result of aberrant genetic alterations and epigenetic abnormalities (Cheng M. et al., 2019). With increased research into bladder cancer and improvements in immunotherapy, methods including intravenous BCG and immune checkpoint inhibitors can influence the development of bladder cancer. At the same time, bladder cancer cells can promote the formation of a tumor immune-suppressive microenvironment. If we can modify or modify TME, it is likely to improve the treatment of bladder cancer. Currently, m6A RNA modification, a very abundant modification in TME, largely influences and alters TME and bladder cancer (Crispen and Kusmartsev, 2020). The level of m6A modification increased significantly in bladder cancer tumor tissues.

Moreover, METTL3 expression levels were similarly upregulated compared to paracancerous tissues. METTL3 can affect the AFF4/NF-κB/myc signaling network in an m6A-dependent manner, promoting bladder cancer progression (Alarcon et al., 2015). Briefly, low METTL3 expression inhibits the *in vivo* proliferation of bladder cancer and, conversely, METTL3 overexpression promotes its proliferation *in vivo*. Briefly, common METTL3 expression inhibits the *in vivo* proliferation of bladder cancer and, conversely, METTL3 overexpression promotes its proliferation *in vivo* (Cheng M. et al., 2019). The mechanism is that METTL3 interacts with the microprocessor protein Dgcr8 to complete miR221/222 processing, which rescues METTL3-induced proliferation of bladder cancer cells, and its processing exerts an oncogenic effect by positively regulating the pri-miR221/222 process in an m6A-dependent manner (Gu et al., 2016).

### Breast Cancer

Breast cancer is the most common malignancy in women and has the second-highest mortality rate in the world (Ahn et al., 2016). Although we have made significant progress in the diagnosis, surgery, and drug development of breast cancer, drug resistance in metastatic cancers remains an insurmountable problem. As research has progressed, we have focused not only on the anti-cancer treatment of tumor cells but also on the TME where the tumor cells reside (Houthuijzen and Jonkers, 2018). Some studies have shown that breast cancer is associated with molecular heterogeneity (Schnitt, 2010). ROS levels in breast cancer cells are associated with the expression and activity of the transcription factor AHR, which controls tumor growth and chemokine production in the TME, and regulating the TME can influence tumor progression (Kubli et al., 2019). The m6A RNA modification in breast cancer TME has a profound impact on the development of breast cancer. In breast cancer, increased expression of METTL14 enhances m6A RNA modifications and has-miR-146a-5p expression, ultimately enhancing breast cancer cell invasion and migration (Yi et al., 2020).

Meanwhile, as an eraser, FTO also plays a vital role in m6A RNA modification. For example, FTO can regulate the migration and invasion of breast cancer cells by regulating the FTO/Mir-181B-3P/ARL5B signaling pathway, which ultimately affects the development of breast cancer. In conclusion, a good grasp of the relationship between m6A RNA modification, TME, and breast cancer will open a new door for breast cancer treatment (Xu et al., 2020).

### Pancreatic Cancer

Pancreatic cancer is cancer with a high mortality rate in the world today. Its late onset makes early diagnosis difficult, and it is often diagnosed late in the patient’s life (Cheng et al., 2017). Although we have made significant advances in the genetics and biology of pancreatic cancer, the results in prolonging the survival time of pancreatic cancer are still not substantial (Ren et al., 2018). In pancreatic cancer, the stability of TME refers to a complex balance between pro-and anti-tumor components (Melstrom et al., 2017). We are combined with clinical trials showing that TME in pancreatic cancer results from the interaction between pancreatic epithelial and mesenchymal cells, which influences the efficacy of radiotherapy, chemotherapy, and immunotherapy. Two distinguishing features of TME in pancreatic cancer are extensive immunosuppression and intensive adhesion formation, accelerating the proliferation of tumor cells (Neesse et al., 2015). m6A RNA modifications also play an essential role in pancreatic cancer, with METTL3 promoting chemo- and radiation resistance in pancreatic cancer cells (Taketo et al., 2018). In TME, ALKBH5 overexpression can inhibit pancreatic cancer by decreasing the level of m6A RNA modification of WIF-1, blocking the activation of Wnt signaling, and increasing the sensitivity of pancreatic cancer cells to drugs (Tang et al., 2020). Meanwhile, ALKBH5 could demethylate the long non-coding RNA KCNK15-AS1 and inhibit pancreatic carcinogenesis. In addition, aberrant activation of the Wnt signaling pathway is also a critical factor in the development and progression of pancreatic cancer, directly or indirectly affecting tumor cells’ proliferation and drug resistance (Morris et al., 2010; Bailey et al., 2016).

## The Application of M6A RNA Modification in Cancer

m6A RNA modifications are among the most abundant chemical modifications within RNA, and we can now exploit their range of properties in RNA, such as abundance, to explore their potential function in cancer therapy (Liu X. et al., 2019). Below we summarize the present and future applications of m6A RNA modifications and the difficulties we have encountered.

## Current Clinical Applications of M6A RNA Modifications

### m6A RNA Modification Might a Biomarker in Cancer

A growing number of studies have shown that m6A RNA modification has excellent cancer diagnosis and prognosis potential (Wu et al., 2020). It relies mainly on the m6A modifying enzyme complex and plays a vital role in the biological processes of cancer cell proliferation, invasion, and migration (Wang Q. et al., 2018). Many experimental and clinical results show that m6A RNA modifications are closely related to the clinical characteristics of patients and show great potential in the diagnosis of tumors as diagnostic biomarkers for human cancers (Meng et al., 2017). For example, the expression level of m6A is significantly higher in circulating tumor cells than in whole blood cells. Suppose the expression level of m6A changed in circulating tumor cells. In that case, the significance of tumorigenesis and progression can be known and used to predict the diagnosis and prognosis of patients (Liu et al., 2020a).

In patients with hepatocellular carcinoma, upregulation of METTL3 and YTHDF expression decreases patient survival (Ma et al., 2017). Patients in the low METTL3/YTHDF1 group have a better prognosis, so the combination of METTL3 and YTHDF1 serves as a biomarker reflecting the malignancy and prognostic outcome of hepatocellular carcinoma. In gastric cancer, abnormal expression of FTO correlates with the progression and metastasis of gastric cancer. The presentation of FTO plays a vital role in promoting the development of gastric cancer. Hence, FTO is an important molecular marker for the diagnosis and prognosis of gastric cancer. In pancreatic cancer, ALKBH also has an important prognostic molecular marker (Yue et al., 2019).

### m6A RNA Modification Might Be a Therapeutic Target in Cancer

In addition to being a biomarker for cancer, m6A RNA modifications have great potential in cancer therapy. Radiotherapy and chemotherapy remain the main treatments for cancer patients, but in recent studies, m6A RNA modifications have shown great potential in cancer therapy (Poller et al., 2018). Analyzing the relevant TCGA dataset, we found that aberrant expression of m6A associated with TP53 gene mutations in AML patients and that m6A mutations reduced survival in AML patients. YTHDF1 is an essential component of the m6A modifying enzyme complex. Its deletion significantly improves the therapeutic effect of PD-L1 checkpoint blockade, revealing that it may be a molecular target for anti-cancer immunotherapy. In addition, m6A RNA modification may also serve as an essential indicator of patients’ sensitivity to radiotherapy. For example, METTL3 promoted resistance to radiotherapy and reduced survival in pancreatic cancer patients, while FTO was expressed at significantly higher levels in CSCC tissues than in pre-cancerous tissues (Liu et al., 2020b). Also, FTO promoted resistance to radiotherapy *in vivo* and *in vitro* by reducing the levels of its target m6A RNA modifications. In summary, m6A RNA modification has the potential to be an effective potential therapeutic target for cancer therapy (Wouters and Delwel, 2016; Yang et al., 2017).

### Future Clinical Applications of m6A RNA Modifications

In recent years, a growing number of studies have demonstrated the ability of m6A RNA modifications to act as diagnostic biomarkers and therapeutic targets. Based on these studies, we should aim to understand and apply them to clinical treatment more fully. For example, the main clinical treatments for cancer are still chemotherapy and radiotherapy, so drug resistance is an inevitable problem. Since m6A RNA modifications are associated with drug resistance, we can link radiotherapy and m6A RNA modifications to explore a promising therapeutic approach. Secondly, RNA and protein are inextricably linked, and the importance of protein as the main performer of biological functions cannot be overstated. If we affect the relevant biological functions of protein through m6A RNA modification, it may bring us unexpected gains in clinical treatment. At the same time, there are still few readers, writers and erasers identified, and we need more research to deepen our understanding of the biological properties of m6A RNA modifications for human health and disease.

### Challenges in Clinical Application

Although m6A RNA modifications are abundantly present in the TME of RNA, the vast majority have no detectable specific biological function, which makes it difficult to further explore their application in clinical aspects. Secondly, the RNA status varies considerably between individuals or different tissues of the same individual, making it difficult to target specific RNA modifications to cells alone and without guaranteeing therapeutic efficacy. Thirdly, little is known about m6A RNA modification, and further understanding of its regulatory mechanisms or possible biological functions in different cancers is necessary. Similarly, the limited number of readers, writers, and erasers involved in m6A RNA modification has also posed a certain obstacle to our research. Finally, most studies on m6A RNA modification have remained *in vitro* analyses, and translational application of the results remains a great challenge due to the dynamic and complex nature of m6A RNA modification and the organism.

## Conclusion

Tumor microenvironment is a complex dynamic system consisting of immune cells, fibroblasts, and lymphocytes, whose interaction with malignant cells influences the development and progression of the cancer process. At the same time, there are hundreds of chemical modifications in TME, and different chemical modifications affect the cancer development process to different degrees. As the most abundant modifications in TME, m6A RNA modifications, in addition to regulating various biological functions such as metabolism, are also clinically meaningful and have the potential to become biomarkers or targets for intervention in tumor therapy. In conclusion, an in-depth understanding of the relationship between TME and m6A RNA modifications is essential for exploring the expression and regulation of oncogenes.

## Author Contributions

All authors conceptualized, wrote, edited, read, and approved the final manuscript.

## Conflict of Interest

The authors declare that the research was conducted in the absence of any commercial or financial relationships that could be construed as a potential conflict of interest.

## Publisher’s Note

All claims expressed in this article are solely those of the authors and do not necessarily represent those of their affiliated organizations, or those of the publisher, the editors and the reviewers. Any product that may be evaluated in this article, or claim that may be made by its manufacturer, is not guaranteed or endorsed by the publisher.

## Abbreviations

TME: tumor microenvironment
m6A RNA modification: the N6-methyladenosine RNA modification
FTO: obesity-associated protein
METTL3/14: methyltransferase-like 3/14
IGF2BP protein: insulin-like growth factor 2 mRNA-binding protein
HDGF: hepatoma-derived growth factor
FOXO3: forkhead box O3
HCC: hepatocellular carcinoma
NSCLC: non-small cell lung cancer
eIF3b: eukaryotic translation initiation factor 3B
SLE: systemic lupus erythematosus
YTHDF: YTH domain-containing family
FTO: obesity-associated protein
YTHDC1: YTH domain-containing protein 1
ALKBH5: alkB homolog 5
CDCP1: CUB-domain containing protein 1
AML: acute myeloid leukemia
HBXIP: hepatitis B X-interacting protein
WTAP: WT1-associated protein
HNRNPG: heterogeneous nuclear ribonucleoprotein G.
